# Isolation, Extraction, Purification, and Molecular Characterization for Thermostable *α*-Amylase from Locally Isolated *Bacillus* Species in Sudan

**DOI:** 10.1155/2021/6670380

**Published:** 2021-05-24

**Authors:** Maha A. Rakaz, Mohammed O. Hussien, Hanan M. Ibrahim

**Affiliations:** Department of Microbiology, Central Laboratory, Ministry of Higher Education and Scientific Research, P.O. Box 7099, Khartoum, Sudan

## Abstract

The aim of this study was to isolate some soil bacteria strain that produced *α*-amylase and subsequent extraction and purification. One hundred soil samples were collected from different geographical areas in Khartoum State such as north Omdurman, Toti Island, and Soba. Samples were analyzed for starch hydrolyzing bacteria. Among several bacteria isolated, *Bacillus cereus* and *Bacillus licheniformis* were identified as active *α*-amylase producers. Both bacteria showed a large zone of clearance of 20 mm when grown on starch-agar plates. The identity was conducted using biochemical characterization and confirmed by sequencing their 16S-rDNA. The constitutive nature of amylase was proved by amplification of the amylase gene from the genome of *B. licheniformis.* The *α*-amylase activity from the spent medium of *B. cereus* and *B. licheniformis* was optimized at pH 8.0 and temperature of 45°C and 65°C, respectively. The *α*-amylase produced by both bacteria is alkalophilic and thermophilic. The experiments confirmed that *B. licheniformis* can be a good source of amylase for industrial applications in Sudan.

## 1. Introduction

Amylases are starch degrading enzymes that cleave the *α*- 1-4 glucosidal linkage of complex polysaccharides [1]. *α*-Amylase (EC 3.2.11) is endo-hydrolyases that acts on *α*-(1,4)-glucosidic linkages in starch and other related oligo- and polysaccharides, thus causing the release of maltooligosaccharides and glucose on the *α*-anomeric form; *α*-amylases are essential for the conversion of starch into oligosaccharides and are critical for many organisms that use starch as a primary source of energy. *α*-Amylase also has potential applications in a number of industrial processes, such as processes in food, fermentation, and pharmaceutical industries [2, 3]. Selection of the right organism plays a key role in the high yield of desirable enzymes. The development of new microbial strains for enzymes production for industrial purposes using a cheap carbon source and nitrogen source is a continuous process. Two major classes of amylases have been identified in microorganisms, namely, alpha-amylase and glucoamylase [4]. Among various extracellular enzymes, alpha-amylase ranks first in terms of the commercial spectrum of applications of *α*-amylase widened in many sectors such as clinical, medicinal, and analytical chemistry. Starch degrading bacteria are mostly important in food, textile, fermentation, and paper industries. The isolation and manipulation of pure culture of starch degrading microorganism from soil have great importance on the biotechnology field. Thus, isolating and manipulating pure culture from various waste materials has manifold importance for various biotechnology industries. The amylase-producing bacteria such as *Bacillus subtilis*, *B. licheniformis*, *B. amyloliquefaciens*, *B. cereus*, and *B. megaterium* and fungi such as *Aspergillus Niger*, *Penicillium*, *Rhizopus*, *Cephalosporium*, and *Neurospora* are major amylase-producing microorganisms [5]. Microbial amylases have been proved to be an alternative to chemical hydrolysis, and the low yield of the enzyme has always been a problem in the commercial production of amylases [6]. The thermophilic organisms are therefore of special interest as a source of novel thermostable enzymes [7]. The advantages for using thermostable *α*-amylase in the industrial process include the decreased risk of contamination, the increased diffusion rate, and belonging to the genus *Bacillus* for industrial applications such as *B. amyloliquefaciens*, *B. stearothermophilus*, *B. cereus*, and *B. licheniformis* [8].

The aim of this study was to isolate, extract, and purify molecular characterization of thermostable *α*-amylase from locally isolated *Bacillus* species from the soil in Sudan.

## 2. Materials and Methods

### 2.1. Sample Collection

One hundred soil samples were collected from potato fields of Khartoum State: North of Omdurman, Toti Island, and Suba/Sudan. Soil samples were collected from the subsurface of 3 to 4 cm depth with the help of a sterile spatula, then transferred in sterile plastic bags, and brought in aseptic conditions to the laboratory.

### 2.2. Isolation of Bacteria

Isolation of soil bacteria was performed by serial dilution method [9]. One gram of soil sample was serially diluted in sterilized distilled water to get a concentration range from 10^−1^ to 10^−4^. A volume of 0.1 ml of each dilution was transferred aseptically to a nutrient agar plate. The samples were spread uniformly using a glass rod. Then, plates were incubated at 37°C for 24 hr. The bacterial isolates were further subcultured on the respective media in order to obtain a pure culture. Pure isolates were maintained at 4°C for further studies.

### 2.3. Primary Screening of Amylase-Producing Bacteria

Hydrolysis of starch was carried out of 10 gm soluble starch in 100 ml distilled water heated in a water bath until dissolved. 20 ml of this solution was mixed in 100 ml of melted nutrient agar poured into the Petri dish after sterilization. A loop full of fresh bacterial culture was picked up by a sterile needle and stabbed onto the agar plate; after 24 hr of incubation at 37°C, the plate was flooded with dilute iodine solution [10]. Hydrolysis of starch was indicated by a clear zone around the growth and unchanged starch gave blue color around the colony about 2 mm. Upon such a result, the isolates were selected to be used for a quantity determination of the enzyme.

### 2.4. Amylase Production

#### 2.4.1. Fermentation Media

Freshly prepared inocula were used to inoculate the production medium. For the preparation of inocula, a loop full of bacterial isolate was transferred in 50 ml of inocula medium containing (g/L) starch 10, peptone 10, yeast extract 20, KH_2_PO_4_ 0.05, MnCl_2_.4H_2_O 0.015, MgSO_4_.7H_2_O 0.25, CaCl_2_.2H_2_O 0.05, and FeSO_4_.7H_2_O 0.01. The flask was placed on a rotary shaker incubator (Sanyo Gallen Kamp, UK) at a speed of 7000 rpm at 0°C for 15 min. The supernatant was collected and used for amylase estimation.

### 2.5. Identification of Amylase-Producing Bacteria

#### 2.5.1. Cultural Characterization

The isolates were identified by colonies morphology with respect to color, shape, size, nature of colony, and pigmentations.

#### 2.5.2. Microscopic Observation

Bacterial isolates were stained using gram stain and observed under oil immersion at high power (100X) magnification under a light microscope. Endospore staining, capsule staining, and a motility test were performed to observe the general morphology and motility of the cells [11].

#### 2.5.3. Biochemical Characterization

Bacterial isolates were characterized biochemically according to Bergey's Manual [12] using Voges–Proskauer (V. P) test, Citrate utilization test, Oxidase test, and normal saline 6.5%

### 2.6. Molecular Identification

#### 2.6.1. Isolation of Bacterial Genomic DNA

Colonies from a single streak on the agar plate were scraped and suspended in PBS and centrifuged. The pellet obtained was dispersed. 600 *μ*l of cell lyses buffer (Guanidium isothiocyanate, SDS, Tris-EDTA) was added and mixed by inverting the vial for 5 min and incubated for 10 min with gentle mixing till the suspension looked almost transparent. 600 *μ*l of isopropanol was layered on top of this solution. The two layers were mixed gently till white strands of DNA were seen and until the solution is homogenous. The strands of DNA were spooled with the help of a pipette tip and transferred into a new vial. 500 *μ*l of 70% ethanol was added to the spooled DNA. The spooled DNA was spun to precipitate DNA at 10,000 rpm for 10 min, the supernatant was discarded. The pellet was air-dried (without allowing it to dry completely). 50 *μ*l of 1X TE was added and the pellet was suspended (incubated for 5 min at 55–60°C to increase the solubility of genomic DNA).

#### 2.6.2. Amplification of 16S RNA Genes

Amplification of the 16S rRNA gene was performed using the primers sets of 16S rRNA fD1 (5′-AGAGTTTGATCCTGGCTCAG-3′) and rP2 (5′-ACGGCTACCTTGTTACGACTT- 3′) [13]. PCR was carried out in a total volume of 50 *µ*l at 94°C for 5 min followed by 35 cycles (94°C for 30 sec, 58°C for 30 sec, and 72°C for 1.5 min) and a final extension at 72°C for 5 min. Finally, the PCR products were subjected to electrophoresis in 1% agarose gel, stained with ethidium bromide, and visualized under ultraviolet (UV) light. The length of the amplicon was approximately 1.5 kb.

#### 2.6.3. PCR for *α*-Amylase Gene

**Figure 1:**
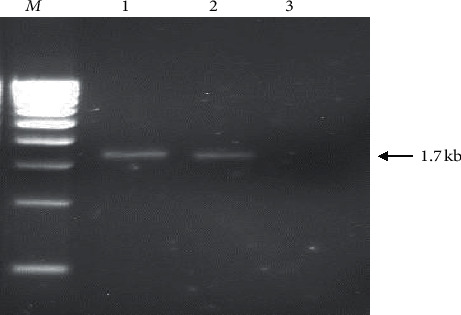
Agarose gel electrophoresis of the products amplified with PCR using the specific primers for *α-*amylase gene. M; 500 bp DNA ladder, Lane 1; *B. licheniformis*, Lane 2; *B. cereus*, Lane 3; negative control.

The isolated DNA samples were amplified using *α*-amylase gene-specific primers; F: 5′-ATTGGTAACTGTATCTCAGCTTGAAGA-3′ and R: 5′-TCCGTCCTCTCTGCTCTTCTATCTT-3′. PCR was conducted in a total volume of 50 *µ*l at 94°C for 5 min followed by 35 cycles (94°C for 30 sec, 58°C for 30 sec, and 72°C for 2 min) and a final extension at 72°C for 7 min. Finally, the PCR products were subjected to electrophoresis in 2% agarose gel, stained with ethidium bromide, and visualized under ultraviolet (UV) light. The length of the amplicon was approximately 1.7 Kb (Figure 1).

#### 2.6.4. 16S rRNA Sequence Analysis for Identification of Bacteria

The amplified DNA was visualized by gel electrophoresis and the PCR products were purified using the solution for Gel Extraction kit (Omega Bio-tek); the purified products were subjected to sequencing by the Sanger technique (3130xl Genetic Analyzer, Applied Biosystems). The most similar bacterial species were found in the GenBank by using BLAST search (http://www.ncbi.nlm.nih.gov/). Neighbor-joining phylogenetic trees were constructed based on 16S rRNA sequences using ClustalW.

### 2.7. Determination of the Optimal Conditions for Amylase Production

#### 2.7.1. Optimum Temperature

The effect of temperature on *α*-amylase activity was studied by adjusting the incubation temperature at 30, 35, 40, 45, 50, 55, 60, 65, 70, and 75°C.

#### 2.7.2. Optimum pH

The synthesis of *α*-amylase was affected by different pH ranges of 3.5 to 5.5 using acetate buffer, pH of 6.0 to 7.5 using phosphate buffer, and Tris-HCl for pH 8.0 to 8.5.

#### 2.7.3. Effect of Reaction Time

Enzyme assays were carried out at different reaction times, namely, 0, 5, 10, 20, 25, and 30 min and it was measured using a spectrophotometer at 540 nm.

#### 2.7.4. Growth Curve

Samples were removed periodically every 6 hr at 0, 6, 12, 18, 24, 30, 36, and 42 hr. The growth curve was measured using a spectrophotometer at 600 nm. The UN inoculated culture broth was used as blank.

### 2.8. Determination of *α*-Amylase Activity

Dinitrosalicylic acid (DNS) method was used to determine *α*-amylase activity as described by Fisher and Stein [14]. UN inoculated culture broth was used as blank. A unit of amylase activity was defined as the amount of amylase required to catalyze the liberation of reducing sugar equivalent to one mol of maltose per minute under the assay conditions. One ml of enzyme sample, one ml of substrate, and 0.5 ml of dinitrosalicylic acid were incubated on a water bath for 15 min, keeping cooling at room temperature and measuring the activity at absorbance 540 nm.

### 2.9. Protein Concentration Assay

Protein was measured by the method of Lowry et al. [15] with bovine serum albumin (BSA) as standard. The concentration of protein during purification studies was calculated from the standard curve. The absorbance at 660 nm was recorded after the reaction and compared with the standard curve plotted against the standard protein (BSA) according to the standard curve.

### 2.10. Purification of Amylase Enzyme

Purification of amylase enzyme was achieved by ammonium sulphate precipitation followed by dialysis. 100 ml of cell-free extract was saturated with centrifuged at 7000 rpm for 15 min. The supernatant was collected and saturated up to 0–30 and 30–80% with ammonium sulphate. Then, the content was centrifuged at 7000 rpm for 15 min and the pellet was collected for further analysis. The enzyme mixture was transferred in a dialysis bag with space size 70 cm and immersed in phosphate buffer pH 7 at 4°C for 24 hr. The buffer was continuously stirred using a magnetic stirrer throughout the process. The buffer was changed three times during the process in order to obtain proper semipurification.

### 2.11. Enzyme Characterization

#### 2.11.1. Thermal Stability

Thermal inactivation of the dextrinogenic activity of the enzyme produced from the bacteria strains was performed by the enzyme incubation at different temperatures for 15 min at ranges of 35, 45, 55, 65, and 75°C.

#### 2.11.2. Storage Inactivation

Storage inactivation of the dextrinogenic activity of the enzyme produced from *B. licheniformis* and *B. cereus* was conducted at temperatures of 4, −20, and −80°C for 24 hr.

#### 2.11.3. pH Stability

*α*-Amylase activity enzyme was tested by different pH ranges of 3.5 to 5.5 using acetate buffer, pH of 6.0 to 7.5 using phosphate buffer, and Tris-HCl for pH 8.0 to 8.5.

## 3. Results

### 3.1. Isolation and Screening of Amylolytic Bacteria

Thirty samples out of 100 (30%) isolates were found to be *Bacillus* sp. Screening of amylolytic bacteria was performed on starch media treated with iodine solution and the colony which showed the disappearance of the blue color around it was selected. In the present study, 66% of the samples showed wide zones around colonies of 3.0, 2.5, 1.5, and 1.25 mm.

### 3.2. Identification of Amylase-Producing Bacteria

#### 3.2.1. Microscopically Characteristics and Biochemical Test

Microscopical examination showed that 66% of isolates were gram positive and positive for motility test, citrate test, growth on normal saline of 6.5%, starch hydrolysis, and VP test. However, the growth at 55°C showed positive for *B. licheniformis* strain and negative for *B. cereus* strain.

### 3.3. Determination of the Optimal Conditions for Amylase Production

#### 3.3.1. Optimum Temperature

**Figure 2:**
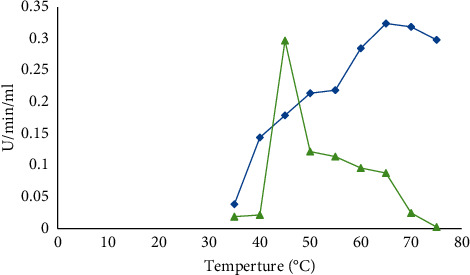
Optimum temperature incubation of *B. licheniformis* (blue) and *B. cereus* (green) for amylase production.

The temperature ranges of 35–45°C were reported to be nominated for the biosynthesis of amylases by *Bacillus* strains [16]. The current results showed that the temperature of 65°C was optimal for *B. licheniformis* while the temperature of 45°C was optimal for *B. cereus* (Figure 2).

#### 3.3.2. Effect of Incubation pH

**Figure 3:**
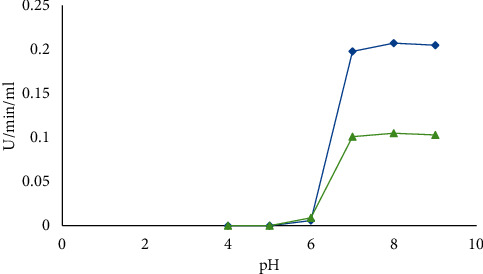
Optimum pH incubation of *B. licheniformis* (blue) and *B. cereus* (green) for amylase production.

The pH ranges from 6.0 to 7.0 have been reported for normal growth and enzyme activity in *Bacillus* strain isolated from soil [17]. In the present study, the production of *α*-amylase by *B. licheniformis* (15.959 U/mL/min) and *B. cereus* (247.20 U/mL/min) was found to be optimum when the initial pH of the fermentation medium was maintained at 8.0. Further increase or decrease in pH resulted in a gradual reduction of amylase production (Figure 3).

#### 3.3.3. Effect of Time Reaction

**Figure 4:**
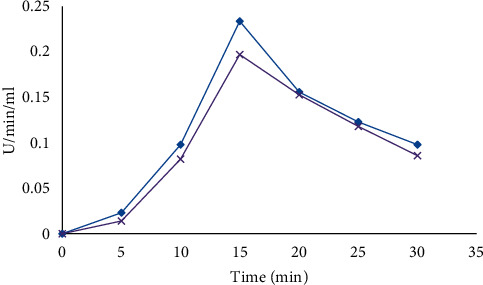
Effect of time reaction of *B. licheniformis* (blue) and *B. cereus* (purple) for amylase production.

Enzyme assays were carried out at different reaction times, namely, 0, 5, 10, 15, 20, 25, and 30 min. The optimum time was found to be at 15 min for enzyme activity assay (Figure 4).

#### 3.3.4. Growth Curve

**Figure 5:**
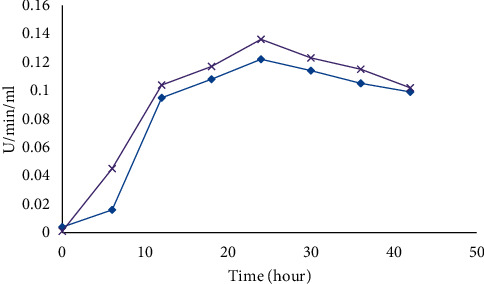
Growth curve of *B. licheniformis* (purple) and *B. cereus* (blue) for amylase production.

Optimization of the incubation period was found to be very critical for maximum production of amylase [18]. In this study, maximum production of amylase for *B. licheniformis* was 0.136 U/mL/min and for *B. cereus* 0.122 U/mL/min at 24 h after inoculation and decreased rapidly thereafter (Figure 5).

### 3.4. Molecular Identification of Amylolytic Bacteria

**Figure 6:**
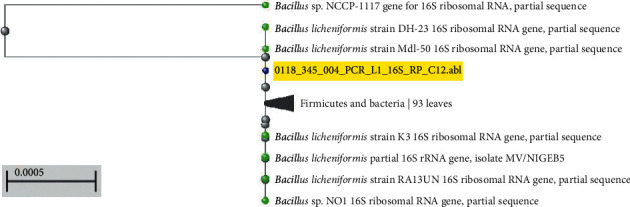
Phylogenetic tree based on partial sequence of 16S ribosomal RNA gene for Sample L1 (highlighted yellow color) showed that the amylase-producing bacteria was *Bacillus licheniformis* based on the first five identities and phylogenic analysis.

**Figure 7:**
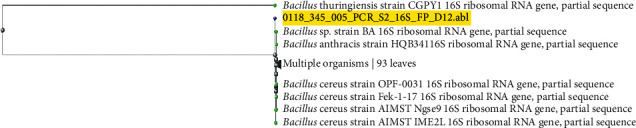
Phylogenetic tree based on partial sequence of 16S ribosomal RNA gene for Sample S2 (highlighted yellow color) showed that the amylase-producing bacteria was *Bacillus cereus* based on the first five identities and phylogenic analysis showed also proximity to *Bacillus thuringiensis*.

On the basis of multiple sequence alignments to construct a phylogenic tree with branch length of 16S rRNA sequence by CLUSTALW, the two isolates exhibited a high level (99-100%) of similarity with the known sequences in NCBI databases was compared using Basic Local Alignment Search Tool (BLAST). Maximum Likelihood (ML) phylogenetic tree for determination of the relationship between our isolates and other reference strains was drawn using MEGA 7.0 software. Phylogenetic analysis indicates that the partial sequence of 16S ribosomal RNA gene of our isolates (L1 and S2) showed that the *α*-amylase-producing bacteria were identified as *Bacillus licheniformis* (accession number LC506177) and *B. cereus* (accession number LC506178), respectively, based on the first five identities (Figures 6 and 7), with nucleotide and amino acid similarities of 99-100%.

### 3.5. Purification of *α*-Amylase Enzyme

In this study, purification of the *α*-amylase enzyme was achieved by ammonium sulphate precipitation followed by the dialysis technique. The activity of the crude *α*-amylase from *Bacillus licheniformis* and *B. cereus* arrived to 247.20 and 15.959 U/ml, respectively, but decreases at each purification step till reaching 8.47 and 0.03 U/ml indicating that the purification step was corrected and enabled to increase specific activity to 5.26 and 0.02 U/mg with purification fold reaching to 0.32 and 0.04 and enzyme recovery arrived at 3.4 and 0.002%, respectively; this decline in activity is imputed to freezing, thawing, and freeze-drying processes (Tables 1 and 2).

### 3.6. Enzyme Characterization

#### 3.6.1. Thermal Stability

**Figure 8:**
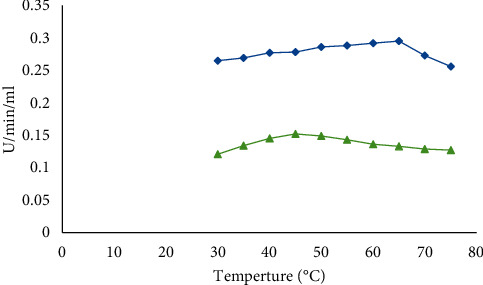
Thermal stability for *B. licheniformis* (blue) and *B. cereus* (green).

The results of thermal stability of the dextrinogenic activity for two *Bacillus s*trains (*B. licheniformis and B. cereus*) in 15 min indicated that the incubation temperature of 45°C is optimum for *B. licheniformis* and *B. cereus* growth. The enzyme was stable for 15 min at a temperature of 45°C while *B. cereus* lost 100% of its activity at 75°C for 15 min but the *B. licheniformis* activity was reduced to 70% at 75°C for 15 min (Figure 8).

#### 3.6.2. Storage Inactivation

**Figure 9:**
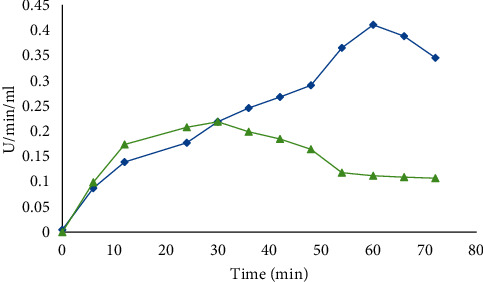
Storage inactivation at 4°C for *B. licheniformis* (blue) and *B. cereus* (green).

Results of storage inactivation of the dextrinogenic activity for *B. licheniformis* and *B. cereus* were found to be with no activity on −80°C for 24 hr. The enzyme was stable for 7 days at a temperature of 4°C for *B. licheniformis* without loss of enzyme activity while for *B. cereus*, the enzyme was stable up to 5 days with only 10% loss in activity (Figure 9).

*B. licheniformis* at −20°C was stored for 2 days with 30% loss in activity but *B.* cereus was stored for 3 hr with 70% loss in amylase activity. At −80°C, both *B. licheniformis* and *B. cereus* amylase activity were lost 100%.

#### 3.6.3. pH Stability

**Figure 10:**
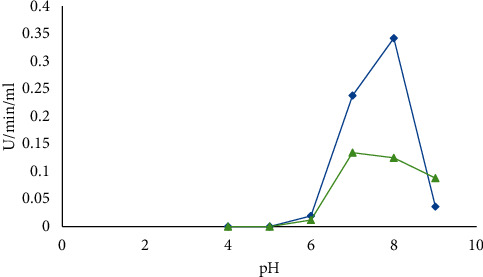
pH stability for *B. licheniformis* (blue) and *B. cereus* (green).

Results indicated that pH of 8.0 was the best for the highest enzyme activity for both *B. cereus* and *B. licheniformis* (Figure 10).

## 4. Discussion

The present study revealed that 65°C and 45°C were optimum temperatures for *α*-amylase produced by *B. licheniformis* and *B. cereus*, respectively. This is in line with Mishra et al. [19] who reported that *B. amyloliquefaciens*, *B. subtilis*, *B. licheniformis*, and *B. stearothermophilus* are among the most commonly used *Bacillus* sp. that produce amylase at temperatures 37–60°C. Our study indicates that optimum *α*-amylase production occurred in pH 8.0 for both *Bacillus* sp. This finding is consistent with the finding of Alkando and Ibrahim [20] who reported that *B. licheniformis* gave maximum *α*-amylase activity of 0.7947 U/mg/ml/min at a pH of 8.0. Furthermore, the present result agrees with those studies reported for *B. licheniformis* [21], *B. subtilis* JS-2004 [22], and *B. Subtilis BI19* [23].

In the current study, the highest production of *α*-amylase for *B. licheniformis* and *B. cereus* was observed at 24 h after inoculation. This may be attributed to the organism which entered the stationary phase and fermentation approached its end point [24, 25]. Maximum studies showed that the production of amylase increased up to the level of 72 h of incubation [26]. Similar results were supported by Abate et al. [27] and Biplab et al. [23]. The current work is more heartening as there was a reduction in the time period that can save energy requirement of the fermentation conditions and provide relatively efficient handling.

The present study indicates that the activity of *α*-amylase produced by *B. licheniformis* was more stable at a high temperature compared to *B. cereus.* A similar finding was observed by Alkando and Ibrahim [20] who reported that *B. licheniformis* lost 72% of its activity at 90°C for10 min. Moreover, for *B. licheniformis,* the amylase enzyme was stable for a week at 4°C without loss of activity unlike *B. cereus* which lost 10% of its activity in the same condition. A similar result was reported by Kumari et al. [28].

## 5. Conclusions

It could be concluded that *Bacillus licheniformis* and *Bacillus cereus* secreted thermophilic and alkaliphilic *α*-amylase under optimized culture conditions. In addition, it was observed that *B. licheniformis* was a better producer than *B. cereus*. The experiments confirmed that *B. licheniformis* can be a good resource of thermophilic and alkaliphilic *α*-amylase for industrial applications in Sudan.

## Figures and Tables

**Table 1:** Partial purification steps of *α-*amylase enzyme from *Bacillus cereus.*

Purification step	Volume	Total protein (mg/ml)	Total activity (U/ml)	Specific activity (U/mg)	Purification fold	Yield%
Crude	82	75.3	15.959	0.211	1	100
(0–30%)	5	4.04	1.617	0.400	1.89	10.13
(30–80%)	25	1.8368	0.0317	0.0172	0.081	0.198

**Table 2:** Partial purification steps of *α*-amylase from *Bacillus licheniformis*.

Purification step	Volume	Total protein (mg/ml)	Total activity (U/ml)	Specific activity (U/mg)	Purification fold	Yield%
Crude	100	52.20	247.20	4.736	1.00	100
(0–30%)	9.5	5.91	13.17	2.228	0.47	5.33
(30–80%)	9.5	8.19	12.31	1.503	0.32	4.98
Dialysis	6.8	1.61	8.47	5.261	1.11	3.43

## Data Availability

The data used to support the findings of this study are available from the corresponding author upon request.
